# Combined Molecular Dynamics and DFT Simulation Study of the Molecular and Polymer Properties of a Catechol-Based Cyclic Oligomer of Polyether Ether Ketone

**DOI:** 10.3390/polym12051054

**Published:** 2020-05-04

**Authors:** Pradeep R. Varadwaj

**Affiliations:** Nanoelectronics Research Institute, National Institute of Advanced Industrial Science and Technology (AIST), Tsukuba Central 1 Chome-1-1 Umezono, Tsukuba 305-8560, Ibaraki Prefecture, Japan; pradeep@t.okayama-u.ac.jp

**Keywords:** thermoplastic polymer, o-PEEK, molecular dynamics and DFT studies, materials and polymer properties, noncovalent interactions, supramolecular assembly

## Abstract

The geometrical, energetic, noncovalent, and material properties of a catechol-based cyclic oligomer of Polyether Ether Ketone (PEEK) called o-PEEK were investigated using Molecular Dynamics (MD) and Density Functional Theory (DFT) simulations. The DFT (and MD) calculation performed with the PBEsol functional (and COMPASS II force field) gave a density of 1.39 (and 1.36) gcm^−3^ and a volume of 2744.5 (and 2808.5) cm^3^ for o-PEEK and are comparable with the corresponding experimental values of 1.328 gcm^−3^ and 2884.6 cm^3^, respectively. The absolute values of the glass transition temperature (*T_g_*) MD simulated using the unit-cell and 2 × 2 × 2 supercell geometries of the o-PEEK system were 424.4 and 428.6 K, respectively. Although these values slightly differ from each other, both are close to the experiment (*T_g_* = 418.2 K). The results of the (charge) density gradient analysis suggest that the supramolecular assembly between the o-PEEK oligomers in the experimentally observed infinite semi-crystal is driven by a wide range of noncovalent interactions. While the individual local interactions between the oligomers were recognized to be weak-to-medium in strength and are theoretically difficult to quantify, the B97-D3/cc-pVTZ level stabilization energy responsible for the formation of each of the five binary complex configurations extracted from the PBEsol relaxed 2 × 2 × 2 supercell geometry of the o-PEEK system was calculated to vary between –3.5 and –33.0 kcal mol^−1^.

## 1. Introduction 

Synthetic development of cheap and lightweight thermoplastic and thermosetting polymer materials is a major goal in the industrial and aircraft engineering fields [1,2,3]. These polymers exhibit attractive material properties. For example, thermosetting polymer resins feature lower processing viscosity [4] and excellent fiber adhesion [5], and have found applications in injection and prepreg manufacture [6], paints [7], and nanocomposite materials [8]. Likewise, their thermoplastic features include high molecular weight [9], excellent thermal insulation ability [10] and high heat resistance [11]. 

Polyether Ether Ketone (PEEK) is a prototypical high-performance heat stable semi-crystalline thermoplastic resin [12]. It has a high melting point, which is around 335 °C [13]. It displays good chemical resistance. Thick samples are generally semi-crystalline and opaque, whereas thin films are usually amorphous and transparent [14]. PEEK also offers very good resistance to abrasion, dynamic fatigue, and radiation [12]. Previous studies have shown that PEEK-based materials are difficult to process and are very expensive [14].

Improvements in the solubility and processability of PEEK have been investigated in several ways [15]. Among these, (1) introduction of a functional group, (2) introduction of a comonomer, (3) disrupting the symmetry by introduction of a meta- or ortho-linked monomer unit, and (4) an increase in the number of ether bonds per repeating unit, are most common [15]. Little theoretical work has been done in the past that reports the physical, chemical, and material properties of the polymer network system [16]. 

In this study, a simple homopolymer of PEEK is considered. It serves here as an archetypal model system for the fundamental physical understanding of any large-scale polymer system. (A homopolymer is a polymer molecule that has one type of chemical repeat unit [17]). The homopolymer is a result of four monomers of PEEK that are repeated within the entire skeletal framework to produce a macromolecule with high molecular weight. It was previously regarded as a (macrocyclic) oligomer of PEEK [18]. We follow the same traditional convention and refer to the oligomer as o-PEEK. We also note that the properties of o-PEEK are very similar to PEEK, [15] and that the 1,2-phenylenedioxy units in it replace hydroquinone residues in the structure of PEEK itself. As this system was experimentally reported some time ago, [18] details of the synthetic strategies, crystallographic information, and glass transition temperature have been known. In specific, it was shown that o-PEEK stabilizes as a semi-crystalline amorphous material. It can be produced by the classical step growth polymerization reaction between catechol and 4,4’-difluorobenzophenone, and is readily soluble in a range of organic solvents.

In this paper, we report the theoretically simulated molecular and polymer properties of o-PEEK, including the electronic structure, intra- and inter-molecular bonding topologies, density, lattice constant, glass transition temperature, and coefficient of thermal expansion. The main purpose of the study is to demonstrate whether Molecular Dynamics (MD) [19] and Density Functional Theory (DFT) [20,21] simulations are appropriate to assess the aforementioned properties of the system. Another purpose is to understand the local stability of the infinite semi-crystal. To this end, we investigate the nature of various intermolecular noncovalent interactions between several oligomeric subunits within the bimolecular approximation. Such interactions are thought to account for the supramolecular assembly between the oligomers, leading to the supramolecular emergence of the semi-crystal system. We apply the reduced density gradient (RDG) based model, [22,23] together with its modified model called Interacting Gradient Model (IGM), [24,25] to the binary systems and carry out an analysis to characterize the reduced density gradient isosuurfaces between various atomic basins computed within the promolecular approximation [24,25,26]. We also analyze the energy stability required to assemble the oligomers. This is expected to provide a means to gain insight into the local strength of the intermolecular interactions involved.

## 2. Computational Details

The experimental unit-cell geometry of o-PEEK [18] was relaxed using periodic DFT calculations. The PBE [27] and PBEsol [28] functionals were used. The Tkatchenko and Scheffler’s ’TS’ correction was invoked to account for the effect of van der Waals dispersion [29]. The k-point meshes 1 × 1 × 1 and 2 × 2 × 2 were used for Brillouin zone sampling. An energy cutoff of 630.0 eV was used, together with the OTGF ultrasoft potential and the Koelling-Harmon scheme for relativistic treatment. The CASTEP software was used [30].

The relaxed unit-cell of o-PEEK and the relaxed 2 × 2 × 2 supercell geometry built using it were supplied for equilibration via MD simulation. To equilibrate the structures, an NVT ensemble simulation at 300 K was performed, followed by an NPT ensemble simulation at 300 K and 1 atm; the equilibrium geometrical properties are listed in Table 1 of the Results and Discussion section. In any case, the Forcite module of the BIOVIA Materials Studio 2019 software package obtained from Dassault Systémes was used [31]. The Ewald representation was used to sum electrostatic interactions with an accuracy < 10 kcal mol^−1^. On the other hand, atom-based representations were used to account for the sum of van der Waals interactions. Andersen’s thermostat (temperature) and the Berendsen pressure control algorithms were used. The COMPASS II Force Field readily available in BIOVIA Materials Studio 2019 was used. Note that the force field general energy function consists of 12 terms including bond (valence) and non-bond interaction terms. The former consists of diagonal (coupling stretch, bending, twisting, out-of-plane potentials) and non-diagonal cross coupling terms (representing interactions between diagonal terms). Similarly, the latter (non-binding interactions) include van der Waals (vdW) and long-range electrostatic interactions. These are described by Lennard-Jones and the Coulombic potential function, respectively. An atom-based summation method with a 12.5 A cut-off radius and a long-range correction was used to calculate the vdW interaction. The Pipeline Pilot Protocol [32] was used to calculate the specific volume, density, enthalpy, and glass transition temperature (*vide infra*), which were evaluated on the equilibrium structures obtained using NVT and NPT dynamics noted above. 

We were interested in the fundamental understanding of the energy strength of the noncovalent interactions between the o-PEEK oligomers of the semi-crystal. To this end, five binary complex arrangements were extracted from the 2 × 2 × 2 supercell geometry of o-PEEK. Each comprises of two homopolymers of o-PEEK. These geometries were then used for single-point calculations in the gas phase. The B97-D3 DFT functional, [33] together with the Grimme-D3(BJ) Dispersion correction [34] and cc-pVTZ basis set, was used. Tight and default algorithms for Self-Consistent-Field convergence and ultrafine integration grid were used. The supermolecular method of Pople [35] was used for the calculation of the stabilization energy, and was corrected for the Basis Set Superposition Error (BSSE) using the counterpoise procedure of Boys and Bernardi [36]. The Gaussian 16 code was used [37].

An interest of this work is to elucidate the nature of intramolecular interactions in describing the geometrical stability of the isolated homopolymer and the nature of the intermolecular interactions occurring between them. The latter are involved in the development of the semi-crystalline amorphous system. Note that there are no direct experimental and theoretical approaches feasible to date to accurately assign the nature of the individual noncovalent interactions in any polymer system. This is because many of them are commingled, making it difficult to identify and quantify each individual contribution. In spite of this, we aimed to provide insight into the nature of these interactions. Thus, we examined the nonlocal |∇*ρ*| behavior of the charge density in the critical binding region, where *ρ* is the charge density. Both the monomeric and binary arrangements of the homopolymer were used. 

Note further that RDG is an innovative approach that has already been employed frequently to reveal noncovalent interactions in chemical systems. It uses the charge density to measure the strength of the interaction in conjunction with the sign of the second curvature of the density λ_2_ to distinguish its nature, where λ_2_ is the second eigenvalue of the Hessian second derivative charge density matrix [22,23,24,25,26]. In other words, the signatures sign(λ_2_) × ρ > 0 and sign(λ_2_) × ρ < 0 represent repulsive (sterically hindered) and attractive interactions, respectively. Because of previous recommendations, [24,25,26] and because the size of the binary systems of o-PEEK under investigation are relatively large (each 272 atoms), we did not reoptimize them further. The RDG isosurfaces analyzed between the interacting atomic domains of the binary systems were obtained using the unit- and super-cell PBEsol geometries. The isosurfaces discussed in the following section are represented by a coloring scheme such that blue, cyan, and green, which refer the level of attraction in descending order. Similarly, the light-yellow and light-brown colored isosurfaces indicate van der Waals interactions, while the red isosurfaces indicate repulsion. The RDG and IGM calculations were performed using combined NCIPLOT [22,38] and in-house codes, and were analyzed with VMD [39].

## 3. Results and Discussions

### 3.1. Lattice Properties

**Figure 1:**
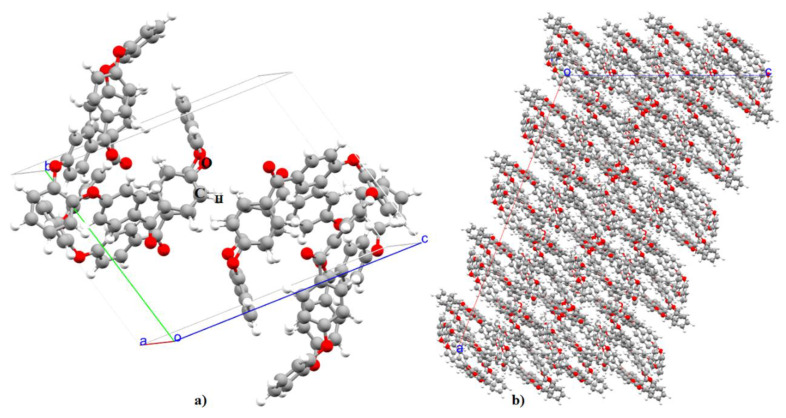
(**a**) The ball-and-stick model of the unit-cell geometry of o-PEEK, obtained using PBEsol. (**b**) The 2 × 2 × 2 supercell model of o-PEEK. Both (**a**) and (**b**) were used for MD simulation of glass transition temperature. Labeling of atom type is shown in (**a**).

Before we dive into the results obtained from this simulation study, we would like to clarify that the unit cell is the basic building block of any infinite (periodic) crystal. This is why the properties of the supercell structures duplicate the properties of the unit-cell. Therefore, most of the results described below were obtained using the relaxed unit-cell of the o-PEEK system and, sometimes, the 2 × 2 × 2 supercell constructed and relaxed using the unit-cell geometry was used to show that the changes in the calculated material properties were not significant. Nevertheless, Figure 1a shows the PBEsol relaxed unit-cell structure of the o-PEEK system; the lattice constants obtained using different approaches are summarized in Table 1. There is considerable agreement between the theoretically calculated and the experimentally reported lattice constants. However, those obtained by DFT are slightly under- or over-estimated compared to experiment; all within 1.3%–6.5%. This change is accompanied by a 4.9% reduction in the cell volume. On the other hand, the lattice constants generated by the COMPASS II force field show good agreement with the experiment, and the degree of mismatch between them is in the range of 0.3%–1.9%. The marginal discrepancy between theory and experiment is not unexpected given that there is a marginal change in the theoretically modelled electronic structure (viz. bond distances and angles) of the o-PEEK system compared to the experiment. 

The packing density ρ_p_ for the unit cell of o-PEEK was calculated to be 1.320 and 1.395 g⋅cm^−3^ with PBEsol. These were evaluated using the 1 × 1 × 1 and 2 × 2 × 2 k-point meshes, respectively. The PBE functional in conjunction with the 1 × 1 × 1 and 2 × 2 × 2 k-point meshes gave ρ_p_ values of 1.267 and 1.340 gcm^−3^, respectively. The MD simulation gave a ρ_p_ of 1.364 gcm^−3^. Clearly, the calculated accuracy compared to the experimental ρ_p_ value of 1.32 g⋅cm^−3^ is (theoretical) model dependent [18]. Even so, these results suggest that the quantitative estimation of ρ_p_ for this type of material using theoretical methods is not far from reality. For PEEK, the experimental densities have been reported to be in the range of 1.26–1.32 gcm^−3^ [40]. 

### 3.2. Polymer Properties

The most important feature of any thermosetting or thermoplastic material is the glass transition temperature, symbolized by *T*_g_. It is this transition temperature at which a polymer goes from the glassy state to a rubbery state upon heating, thus defining the low temperature regime for the mechanical property of polymers [41,42,43,44]. For the calculation of *T*_g_, the PBEsol unit- and 2 × 2 × 2 supercell geometries of o-PEEK were separately relaxed using MD. A dilatometric analysis was then carried out [45,46,47]. This approach provides a clear and unambiguous route for calculating the volume and density for an amorphous lattice, in which, the volume temperature dependence generally leads to a surprisingly good prediction of *T*_g_ [45]. Nevertheless, the approach involves fitting a series of density versus temperature data to an analytical function such that the coefficient of thermal expansion (CTE) can be sigmoidal, where CTE = γ + δ tanh *t*. In this relation, *t* is the reduced temperature with *t* = (*T*-*T*_g_)/*T*_0_, where γ, δ, *T*_g_ and *T*_0_ are the fitting parameters. Upon integration, the volume at the glass temperature (*V*_g_) enters as an additional fit parameter. The details of this procedure are described in the user manual of the Pipeline Pilot Protocol [32] of Materials Studio [31].

Note that the dilatometric analysis incorporates the Fox-Flory’s Free Volume Theory [46,47] of amorphous polymers. The theory empirically relates the volume and density to the glass transition temperature. We thereby examined the change of specific volume with respect to the temperature, as well as the change of density as a function of temperature. As such, these properties of the system were evaluated over the temperature range between 700 and 100 K, and with 30 temperature steps (the number of steps between the starting and ending temperatures). Twelve sets of calculations were performed, starting from two configurations of o-PEEK. Six of these correspond to unit-cell geometry of o-PEEK, and the remaining six sets correspond to the supercell geometry. The sets of data for each geometry were averaged separately. 

**Figure 2:**
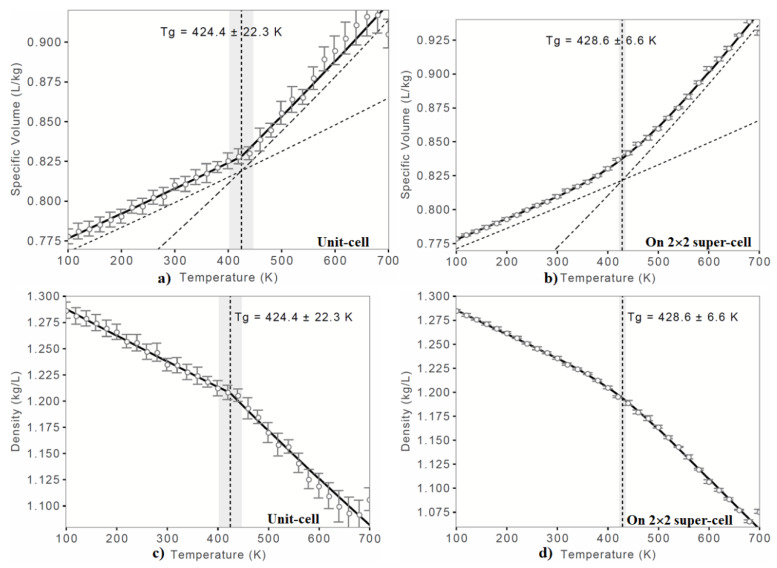
Dilatometric results of o-PEEK, where the equilibrium start- and end-temperatures were 700 K and 100 K, respectively, with the step size of 30 between them. (**a)**–(**b**) Specific volume vs. Temperature and (**c)–**(**d**) Density vs. Temperature. The glass transition temperature (*T*_g_) with standard deviation is shown for each case.

Figure 2a shows a plot of the specific volume as a function of temperature, and Figure 2c shows a plot of the resulting density as a function of temperature, obtained using the unit-cell geometry. The corresponding graphs generated using the supercell geometry of the system are shown in Figure 2b,d, respectively. The small circles in the graph represent the state of the system at a specific temperature and volume/density during simulation. The graphs show that as the temperature rises, the specific volume increases. Concomitant with this, the packing density steadily decreases. The behavior is typical of the volume/density versus temperature profile for many amorphous polymer materials.

From Figure 2a, it is apparent that there is approximately a linear dependence between the specific volume and temperature both above and below the transition temperature, especially when the data of the unit-cell geometry was used for analysis. The kink of the curve is representative of *T*_g_. Because of this behavior, and in general, the data before and after the kink were fitted to two linear equations. The temperature at which the specific volume-temperature gradient, as well as density-temperature gradient, changes is *T*_g_, which corresponds to the slope of the curve. A similar argument is applicable to the data obtained using the supercell geometry. However, the plot in Figure 2c shows that the kink region is not very prominent. This is arguably due to the size of the model geometry and the segment movements, which collectively control the degree to which the cooling effect determines the *T*_g_ profile. 

The changes in the slope of both the curves shown in Figure 2a,c were 424.4 ± 22.3 K, regardless of whether the volume-temperature profile or the density-temperature profile were used. As noted above, and when the 2 × 2 × 2 supercell geometry was used, both the density and volume were slightly affected. Therefore, the slopes of the corresponding curves shown in Figure 2b,d were changed and the resulting *T*_g_ values were estimated to be 428.6 ± 6.6 K. 

The marginal difference in the absolute *T*_g_ values calculated using the unit- and super-cell models is not unexpected since the MD-based equilibrium process is intrinsically stochastic and that *T*_g_ is packing dependent. In any case, the simulated *T*_g_ values were close to the experimental value of 418.2 K (145 °C) reported both for PEEK [15] and o-PEEK [15,18]. While the experimental accuracy of *T*_g_ is unknown, the absolute value of simulated *T*_g_ is about 10 K higher than that reported experimentally. 

**Figure 3:**
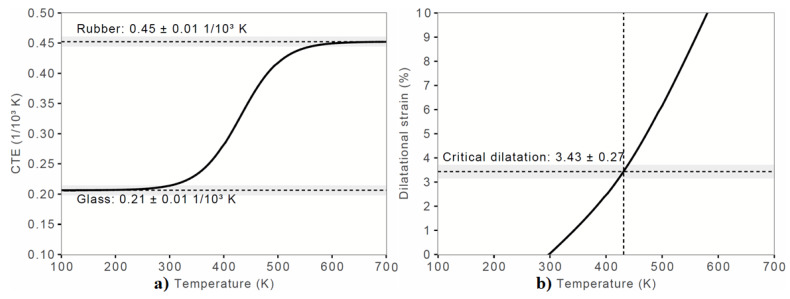
Dilatometric analysis: (**a**) CTE vs Temperature; and (**b**) Dilatational strain vs Temperature. The unit-cell geometry of o-PEEK was used for MD simulation, with the starting and ending temperatures for equilibration being 700 and 100 K, respectively, with the step size of 30 between them.

Figure 3a shows the nature of the CTE as a function of temperature. It represents a pseudo-second order transition. Since the CTE is a measure of the expansion or contraction of a material as a result of changes in temperature [45,48], the pseudo S-shaped nature of the plot suggests that the material should contract upon decreasing the temperature from 600 to 300 K, and this behavior is typical of the glass transition region when cooling from the supercooled melt to glass [48,49]. Nevertheless, the CTE is found to be positive, and is different above and below *T*_g_. It is equal to 0.21 ± 0.01 × 10^−3^ K^−1^ when the material behaves like a glass and equal to 0.45 ± 0.01 × 10^−3^ K^−1^ in the rubber state. The values suggest that the CTE of the material in the glassy state is almost double compared to that of the rubbery state. In addition, the critical dilation for the material was found to be 3.43 ± 0.27 (Figure 3b). The corresponding values obtained with the 2 × 2 × 2 supercell model of o-PEEK were 0.23 ± 0.02 × 10^−3^ K^−1^, 0.47 ± 0.02 × 10^−3^ K^−1^ and 3.46 ± 0.20, respectively, showing a very marginal effect of cell size on the properties of o-PEEK. 

### 3.3. Nature of Noncovalent Interactions and Complex Stabilities

**Figure 4:**
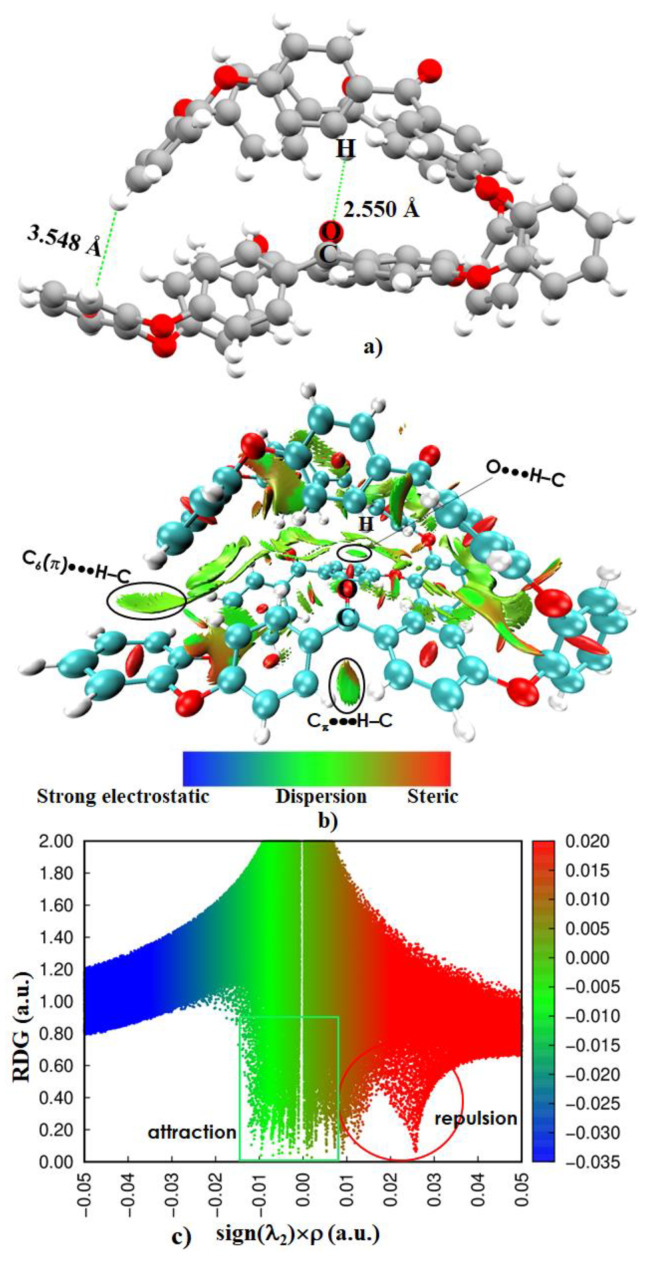
(**a**) The B97-D3 relaxed ball-and-stick model of the o-PEEK oligomer. (**b**) The RDG isosurface plot (0.6 a.u.) and (**c**) the sign(λ_2_)×ρ vs. RDG plot, showing the importance of intramolecular interactions in driving the structural stability of the homopolymer. Selected intramolecular contact distances are shown in (**a**) and some of these are marked in (**b**). Labeling of atom type is shown both in (**a**) and (**b**). The blue and green colors in (**b**) indicate electrostatically and dispersion dominant interactions, respectively. On the other hand, red indicates repulsion.

Figure 4a,b show the energy-minimized geometry and the RDG-based isosurface plot of the o-PEEK oligomer. The intramolecular bonding topologies that can be deduced from the isosurface plot in 4b are very complex as can be anticipated for any large-scale supramolecular or polymer systems. 

Several notable bonding features are apparent from the isosurface plot shown in Figure 4b. The most prominent ones responsible for holding the various fragments of PEEK within the domain of the isolated o-PEEK system are O···H(C), C_π_···H(C), (C=C)_π_ ···H(C), C_6_(π)···H(C) and π···π. The O atom of the ketone fragment C=O facing the interior framework of o-PEEK forms four O···H(C) hydrogen bonds and one of these is marked in (a) – (b) of Figure 4. Of the four, two are relatively shorter than the other two, with the O···H(C) bond distance pairs 2.550 and 3.201 Å. These are the result of the orbital involvement between the antibonding σ* orbital of the C–H fragments and the lone-pair bonding orbitals of the O atom of the C=O fragment. They are relatively localized interactions compared to C_π_···H(C), (C=C)_π_···H(C), C_6_(π)···H(C) and π···π. 

On the contrary, the C_π_···H(C), (C=C)_π_···H(C) and C_6_(π)···H(C) interactions are a result of interaction between the anti-bonding σ* orbitals of the C–H fragment and the bonding π-orbitals of the C, C=C and the C_6_ fragments, respectively. 

The delocalized nature of the reduced density gradient domains observed within the interior framework of the oligomer, evidenced by the irregularly shaped isosurfaces in green, indicates that these interactions are long-ranged, weak-to-medium strength, well-dispersed, and sometimes localized. This is also consistent with the sign(λ_2_) × ρ vs. RDG plot shown in Figure 4c) in which –0.0015 a.u. < sign(λ_2_) × ρ < +0.0080 a.u. The brownish-red isosurfaces indicative of Figure 4b may represent semi-repulsive interactions, appearing as a result of significant steric crowding between the adjacent fragments of the o-PEEK system. All these results suggest that the interior of o-PEEK constitutes an adhesive-like bonding synthon that coordinates the packing between the PEEK fragments, thereby effectuating the geometry of the homopolymer to fold. This view may be consistent with an experimental study,^18^ demonstrating that the cyclic tetramer adopts a highly convoluted and quite unprecedented structure in which the ortho-substituted catechol residues result in the chain wrapping sharply back and forth across the molecule, leading to a tightly folded conformation somewhat resembling a small globular protein. The author recognizes that there is no straightforward theoretical protocol viable to accurately quantify the energy of each of the intramolecular interactions in o-PEEK.

**Figure 5:**
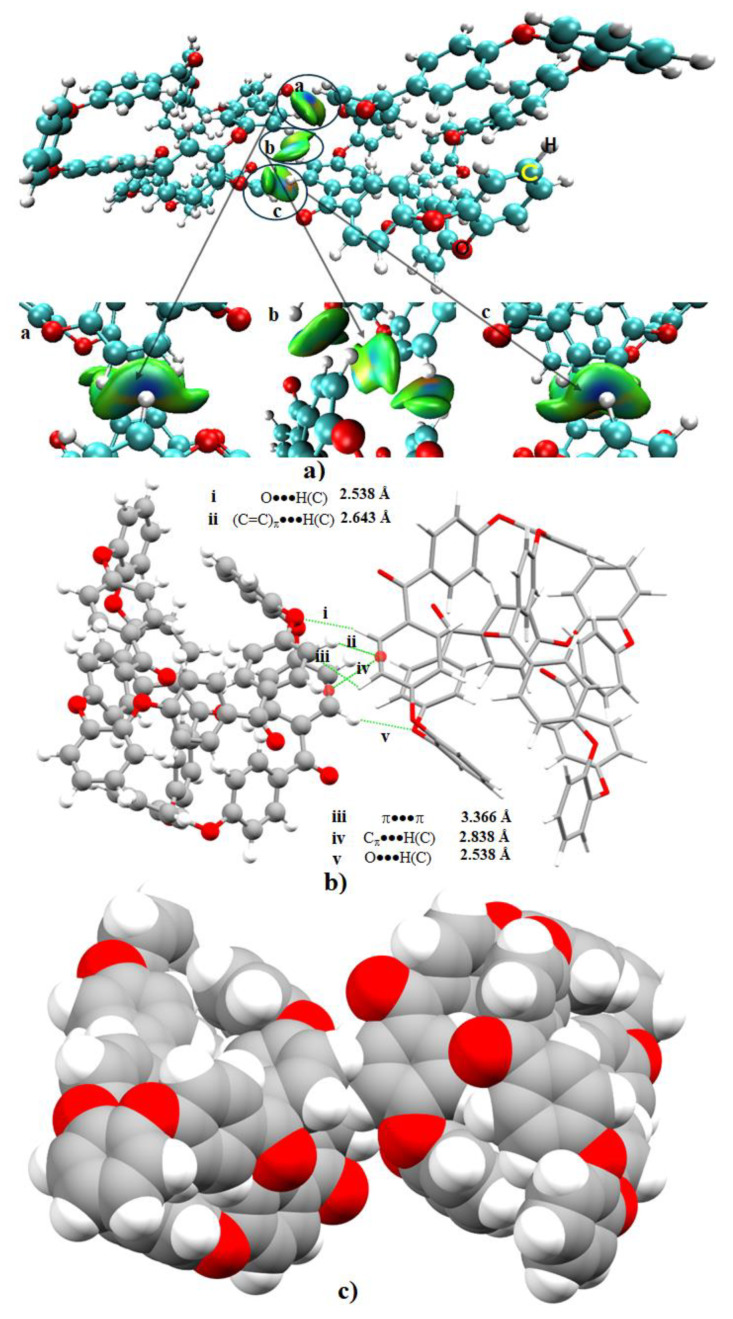
(**a**) The result of a modified RDG-based isosurface (0.005 a.u.) analysis for the (o-PEEK)_2_ complex. (**b**) The ball-stick-tube composite model of the corresponding system, showing the presence of possible intermolecular interaction distances that are marked by i)-v). (**c**) A space-filling model, showing the overlap between close-lying atomic domains.

Due to the relatively large size of o-PEEK’s binary systems, the IGM approach was used to analyze the nature of the reduced charge density topologies. Figure 5a represents the corresponding results of a binary complex geometry of o-PEEK. It was extracted from the unit-cell of o-PEEK (Figure 1a) by removing the periodic boundary conditions. The promolecular approximation was utilized [24,26]. In this approximation, the atomic electron densities are summed up, but the associated atomic gradients are not allowed to interfere. The isosurfaces enclose the bond critical points and also accounting for in extracting the desired interaction present in |∇*ρ*|. In any case, the intermolecular region in the binary complex shown in Figure 5a comprises of three individual isosurfaces. These are marked by a, b and c; the local images corresponding to these isosurfaces are magnified at the bottom of Figure 5a.

Isosurface a exhibits the combined effect of three interaction types, and is equivalent to isosurface c due to symmetry. The irregular greenish regions corresponding to the left and right portions of each isosurface represent the C_π_···H(C) and O···H(C) interactions, respectively, whereas the intermediate bluish-green semicircular portion of the same isosurface corresponds to (C=C)_π_···H(C). Similarly, isosurface b is the combined effects of π···π stacking (parallel displaced) and C_π_···H(C) contacts. The former is the consequence of attraction between the π-electron densities associated with the C=C double bonds of the aromatic rings of the two oligomers that are in close proximity. The latter is due to the attraction between the π cloud on the C atoms of the C=C bond in one oligomer and the H atom of the second interacting oligomer. Noncovalent bonding interactions of this kind are common in chemical systems where aromatic and/or non-aromatic fragments are in close proximity to each other, such as benzene dimers, epoxy composites, and similar systems that comprise Lewis bases of different types [22,23,24,25,26,38,50,51,52,53,54,55,56].

Figure 5b summarizes the intermolecular distances associated with the contacts in (o-PEEK)_2_. Among these, the *r*(O···H) is the shortest of 2.538 Å. This contact is directional (∠O···H–C = 161.3^o^) and localized. One might presume that this could be stronger than the (C=C)_π_···H(C) contact observed in the same system. The rationale for this is that the distance between the mid of the (C=C)_π_ bond and the bonded H atom is 2.643 Å, a distance longer than the corresponding *r*(O···H) distance. However, it should be remembered that the interaction between (C=C)_π_ and H(C) is largely delocalized over the C=C double bond, as manifested by the isosurface being more bluish than green. This explains why the (C=C)_π_···H(C) interaction can be considered to be relatively stronger than that of the O···H hydrogen bond. 

The intermolecular distances for π···π and C_π_···H(C) interactions representing isosurface b are 3.366 and 2.838 Å, respectively. This indicates that the former is somewhat weaker than the latter. The space-filling model shown in Figure 5c suggests that the molecular fragments susceptible to intermolecular interactions overlap, providing further evidence that the oligomers are linked to one another. 

From the extent of charge density delocalization in the bonding region that determines the color of the isosurface in Figure 5a, it can be concluded that the strength of the intermolecular interaction in the (o-PEEK)_2_ system may follow the trend: (C=C)_π_ ···H(C) > O···H(C) > C_π_···H(C) > π···π. This trend in the intermolecular bonding stability is somehow concordant with the intermolecular distances associated with these interactions. The O···H hydrogen bonding interactions revealed in this study were previously shown to represent a significant contribution to a protein conformation [57].

**Figure 6:**
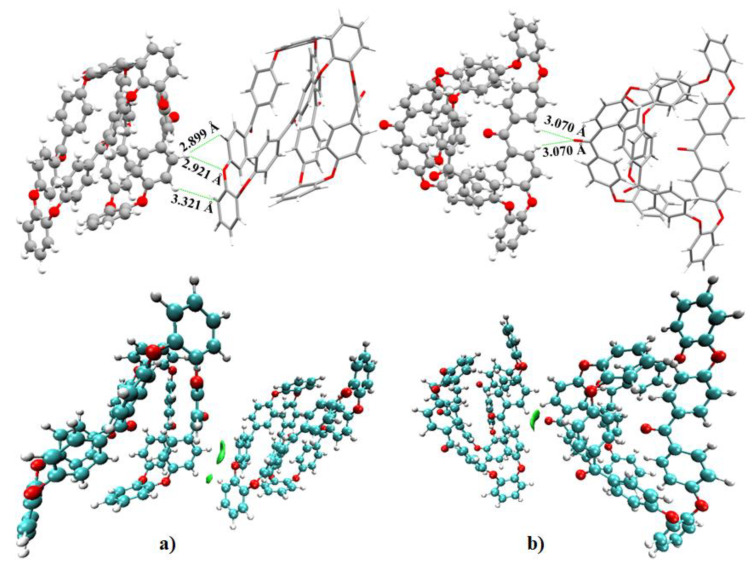
(**a**)–(**b**) (Top) The intermoelcular contact geometries and (Bottom) the IGM isosurface plots (0.005 a.u.) for two randomly selected binary complexes of o-PEEK. Selected intermolecular distances are shown.

**Figure 7:**
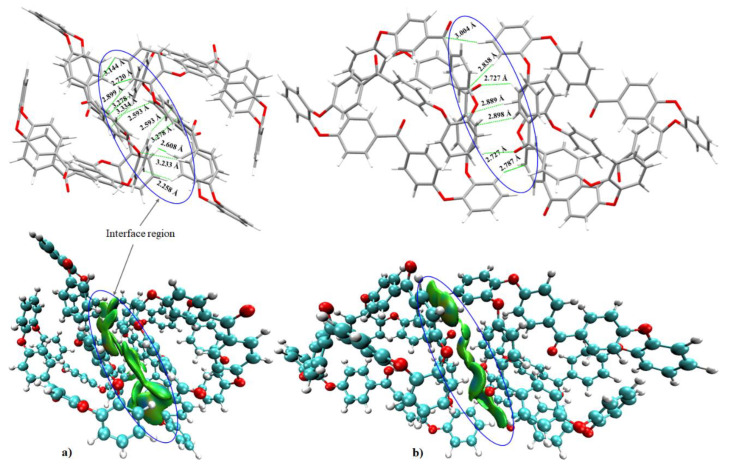
(**a**)–(**b**) (Top) The intermoelcular contact geometries and (Bottom) the IGM isosurface plots (0.005 a.u.) of two randomly selected binary complexes of o-PEEK. Selected intermolecular distances are shown.

The uncorrected and BSSE corrected stabilization energies calculated for (o-PEEK)_2_ are –8.86 and –7.65 kcal mol^−1^, respectively. This implies that the strength of the individual interactions in (o-PEEK)_2_ is indeed weak, yet significant for the supramolecular assembly. This view is nontrivial since the medium strength dimerization energy [46] calculated for the entire system is the sum of all individual interactions involved. Perutz has previously demonstrated that the link between the proton donors and the π electrons of the benzene is typically described by a bond energy between 2 and 4 kcal mol^−1^, a value large enough to be biologically significant [55]. Such contacts are the most frequently occurring intermolecular interactions between the oligomers. However, these are just not the only ones that determine the packing between the oligomers in the entire semi-crystal. To discern the credibility of this hypothesis, another four conformationally different (o-PEEK)_2_ systems were randomly extracted from the supercell geometry shown in Figure 1b. An IGM analysis was performed on each of these geometries. The results are illustrated in Figure 6 and Figure 7. 

From the intermolecular geometry shown in Figure 6a, it is obvious that there are three local contacts between the two o-PEEK oligomers in (o-PEEK)_2_. These include O···H(C), (C)H···H(C), and C_π_···H(C). The bond distances associated with these contacts are 2.889, 2.921 and 3.321 Å, respectively. The isosurfaces corresponding to the three contacts are localized (see bottom of Figure 6a), suggesting the presence of weak interactions. 

(o-PEEK)_2_ in Figure 6b comprises of two O···H(C) contacts. These are equivalent (*r*(O···H(C)) = 3.070 Å each). This result shows that the strength of the O···H(C) interaction is different in going from one binary complex to the other, which is a frequently occurring interaction controlling the supramolecular assembly between the o-PEEK oligomers of the semi-crystal. The BSSE corrected stabilization energy for (a) and (b) of Figure 6 are –3.85 and –6.18 kcal mol^−1^, respectively. 

By contrast, the BSSE corrected stabilization energies for complexes (a) and (b) of Figure 7 were –32.61 and –31.18 kcal mol^−1^, respectively. These are most stable in the series of five binary complexes tested. Even so, neither of these two complex systems constitutes the unit-cell geometry of the crystal (Figure 1a). Their vitality is revealed only when the unit-cell is expanded. This suggests that the unit-cell geometry cannot always be regarded as an exemplar to understand the details of the local nature of the geometrical and energetic stabilities of any large-scale system.

The large stabilization energy for each of the two complexes in Figure 7 is not very surprising. The two oligomers in each system are coupled with each other via multi-fold intermolecular interaction topologies. These are long-ranged and well-dispersed, as demonstrated by the IGM isosurfaces shown at the bottom of Figure 7. The O···H(C), (C=C)_π_···H(C) and C_π_···H(C) contacts are common to both (a) and (b). However, the (C)H···H(C) contacts are prominent in the former and the (C=C)_π_···H(C) and (C_6_)_π_···H(C) contacts are prominent in the latter, in agreement with the spread in the isosurface topology that appears in the intermolecular (interfacial) region.

**Figure 8:**
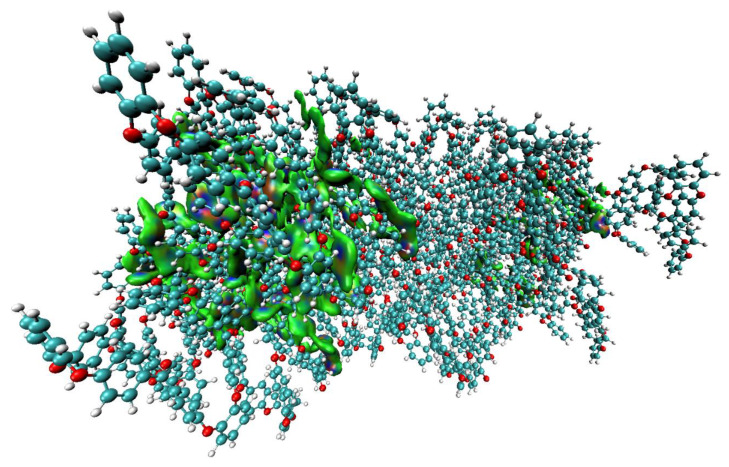
Illustration of dispersed IGM isosurface domains (0.002 a.u.) between 20 randomly selected oligomers of o-PEEK, accountable for the emergence of the semi-crystal. Each oligomer comprises of 136 atoms, and that there are 40 oligomers in the geometry of the 2 × 2 × 2 supercell (5440 atoms).

Figure 8 shows the dispersed nature of the IGM isosurface involved in retaining the oligomers in the semi-crystal, thereby revealing the crucial importance of noncovalent interactions in the supramolecular assembly. Although the structure used here originates from the experimental geometry of the system, the PBEsol and COMPASS II force field relaxed geometries predict substantially similar results.

## 4. Conclusions

In this study, we examined the electronic structure, energetic, noncovalent, and polymer properties of the o-PEEK homopolymer using MD and DFT simulations. We conclude the following:

(i) MD and DFT simulations results suggest that the packing density, volume, and lattice constant of o-PEEK predicted using these methods are not only consistent with each other but also with the experiment.
(ii) COMPASS II force field coupled MD simulations could adequately predict the *T_g_* of the o-PEEK system close to the experiment. This was true regardless of the size of the semi-crystalline system examined. The result also suggested that the glassy state of the semi-crystalline polymer system might be explained using relatively small-scale MD simulations.
(iii) The compactness of the structure of the o-PEEK oligomer was recognized to be driven by the vast number of intramolecular interactions. These were identified and characterized to be O···H(C), C_π_···H(C), (C=C)_π_···H(C), C_6_(π)···H(C) and π···π.
(iv) It was found that the local spatial arrangement between o-PEEK oligomers in the semi-crystalline system is controlled by a number of coordination modes. These modes appeared in various flavors, including intermolecular contacts composed of O···H(C), (C=C)_π_···H(C), C_π_···H(C), (C)H···H(C), (C=C)_π_···O(C), and (C_6_)_π_···H(C). Clearly, the stability of any of the five binary systems examined is controlled not only by the degree to which the energy of any individual interaction dominates, but also by the number of various such intermolecular interactions involved.
(v) Analysis of the stabilization energies of the binary complex models suggested that the overall energy strengths would vary between –3.85 and –32.61 kcal mol^−1^ and are controlled by the nature of the spatial arrangement between the oligomers. The energy of each complex system was realized not simply by a single interaction between the oligomers, but was rather a collection of several contacts that determine overall stability.

## Figures and Tables

**Table 1:** Comparision of selected DFT-PBESol and MD (COMPASS II) calculated unit-cell properties of o-PEEK with experiment. ^a.^

Property	DFT ^b^	MD ^b^	Expt. ^c^	% Change(DFT) ^d^	% Change(MD) ^d^
ρ_p_(g/cm^3^)	1.395	1.364	1.328	+5.1	+2.2
*V*(cm^3^)	2744.5	2808.5	2884.6	–4.9	–2.6
***N***	272	272	272		
Lattice Constants					
*a*/Å	14.142	14.281	14.328	–1.3	–0.3
*b*/Å	14.142	14.281	14.328	–1.3	–0.3
*c*/Å	16.463	16.612	17.525	–6.5	–5.5
α/deg	107.7	107.9	110.5	–2.6	–2.4
β/deg	107.7	107.9	110.5	–2.6	–2.4
γ/deg	63.2	62.6	61.4	+2.8	+1.9
	Glass Transition Temperature ^c^				
***T_g_***/K		424.4 ± 22.3	418.2		

^a^ Properties include the packing density (ρ_p_), the unit-cell volume (*V*), the number of atoms in the unit-cell (*N*), the lattice constants (*a*, *b*, *c*, α, β and γ) and the glass transition temperature (*T_g_*). ^b^ This work. The PBESol calculation was performed with the k-point mesh 2 × 2 × 2. ^c^ Experimental values were taken from [18]. *T_g_* was calculated using MD simulation and the DFT relaxed unit-cell structure of o-PEEK was supplied. ^d^ The positive and negative signs indicate the percentage of increase and decrease of a specific calculated property compared to experiment, respectively.
